# Needle Arthroscopic Inspection and Treatment of (Osteo)chondral Lesions of the Ankle in Unstable Syndesmotic Injuries Treated With Suture Button Fixation: A Standardized Approach

**DOI:** 10.1016/j.eats.2023.02.050

**Published:** 2023-06-12

**Authors:** Alex B. Walinga, Jari Dahmen, Tobias Stornebrink, Gino M.M.J. Kerkhoffs

**Affiliations:** a Amsterdam UMC location University of Amsterdam, Department of Orthopedic Surgery and Sports Medicine, Amsterdam, The Netherlands; b Amsterdam Movement Sciences, Musculoskeletal Health, Sport, Amsterdam, The Netherlands; c Academic Center for Evidence-based Sports Medicine (ACES), Amsterdam, The Netherlands; d Amsterdam Collaboration for Health & Safety in Sports (ACHSS) international Olympic Committee (IOC) Research Center, Amsterdam, The Netherlands

## Abstract

Both acute and chronic syndesmotic injuries are associated with (osteo)chondral lesions of the ankle in at least 1 out of 5 patients. Having a common denominator of an acute traumatic injury being external rotation and forced dorsiflexion, the injury of a combined unstable syndesmotic injury and a potentially present concomitant (osteo)chondral lesion to the ankle warrants a thorough diagnostic and interventional approach. Furthermore, early diagnosis may prevent further damage to the joint through lifestyle changes and increase the chances of successful conservative or surgical treatment, potentially reducing the progression toward end-stage osteoarthritis. Consequently, technological advances and financial considerations have led to the development of a minimally invasive needle arthroscopic inspection and treatment of concomitant (osteo)chondral injuries of the ankle in the combined treatment of unstable syndesmotic injuries with suture button fixation. The present Technical Note describes this innovative minimally invasive assessment and concomitant treatment technique.

Syndesmotic injuries can be caused by external rotation and hyperdorsiflexion of the ankle joint. During this trauma mechanism, external rotational forces are impacted on the talar dome and the distal tibia, which potentially results in intra-articular pathologies such as osteocartilaginous damage, loose bodies, or impingement. A recent study showed that acute and chronic syndesmotic injuries are associated with (osteo)chondral lesions ([O]CLs) of the ankle in at least 1 out of 5 patients.^1^^,^^2^ The study concluded that incidence rates of (O)CLs of the ankle were 22% in the acute group and 24% in the chronic syndesmotic injuries group. These findings are of clinical relevance because they show that clinicians should be extremely aware to accurately recognize and concomitantly treat the cartilage damage alongside a potential stabilization of the syndesmotic joint. Minimally-invasive needle arthroscopy is a technology that can improve the effectiveness and practicability of providing a concomitant diagnostic and therapeutic protocol for patients with unstable acute and chronic syndesmotic ankle injuries who may have intra-articular ankle (O)CLs (Table 1). This technology, along with financial considerations, has led to the development of minimally invasive needle arthroscopic inspection and treatment of cartilage injuries in the ankle in combination with suture button fixation for unstable syndesmotic injuries.^3^, ^4^, ^5^, ^6^ In the present Technical Note, we describe a standardized approach to needle arthroscopic diagnosis and concomitant treatment of (osteo)chondral injuries to the ankle in patients undergoing suture button fixation for unstable acute or chronic syndesmotic injuries.

**Table 1:** Indications and Contraindications

Indications
Acute isolated and nonisolated unstable syndesmotic injuries requiring a suture button stabilization/fixation
Chronic isolated and nonisolated unstable syndesmotic injuries requiring a suture button stabilization/fixation
Contraindications
Presence of big osteophytes to the ankle joint preventing an adequate introduction of the instruments, as well as a proper visualization of the joint
Decreased or inadequate vascular status
Infections; peripheral arterial occlusive disease
Critical soft-tissue conditions/compromise
Relative contraindication: previous surgery or surgeries to the ankle joint
Severe joint-space narrowing

## Surgical Technique

The procedure is demonstrated in Video 1 using a step-by-step approach. The technique is demonstrated in the current article using intra-operative videos.

### Equipment

The needle arthroscopic device (NanoScope; Arthrex, Naples, FL) consists of 2 main components: the sterile disposable handpiece set and the portable video console. This handpiece set includes a semi-rigid, 0° needle arthroscope, sharp and blunt obturators, and suitable sheaths.^7^ A 2 or 3 mm shaver (Arthrex) can be used for the debridement of intra-articular pathologies. A suture button (TightRope; Arthrex) is used for the stabilization of the syndesmosis.

### Patient Setup

The patient is positioned in a supine position on a standard operating table, with the foot at the edge of the table. The surface anatomy of the ankle is marked out (i.e., medial malleolus, lateral malleolus, tendon of the tibialis anterior muscle, peroneus tertius tendon, and the superficial peroneal nerve), as well as the portal locations.^7^^,^^8^ A tourniquet is applied at the thigh and is inflated to 250 mm Hg. The surgical field is disinfected with a chlorohexidine solution and covered with sterile draping.

### Portal Placement, Arthroscope Introduction, and Joint Distention

**Fig 1:**
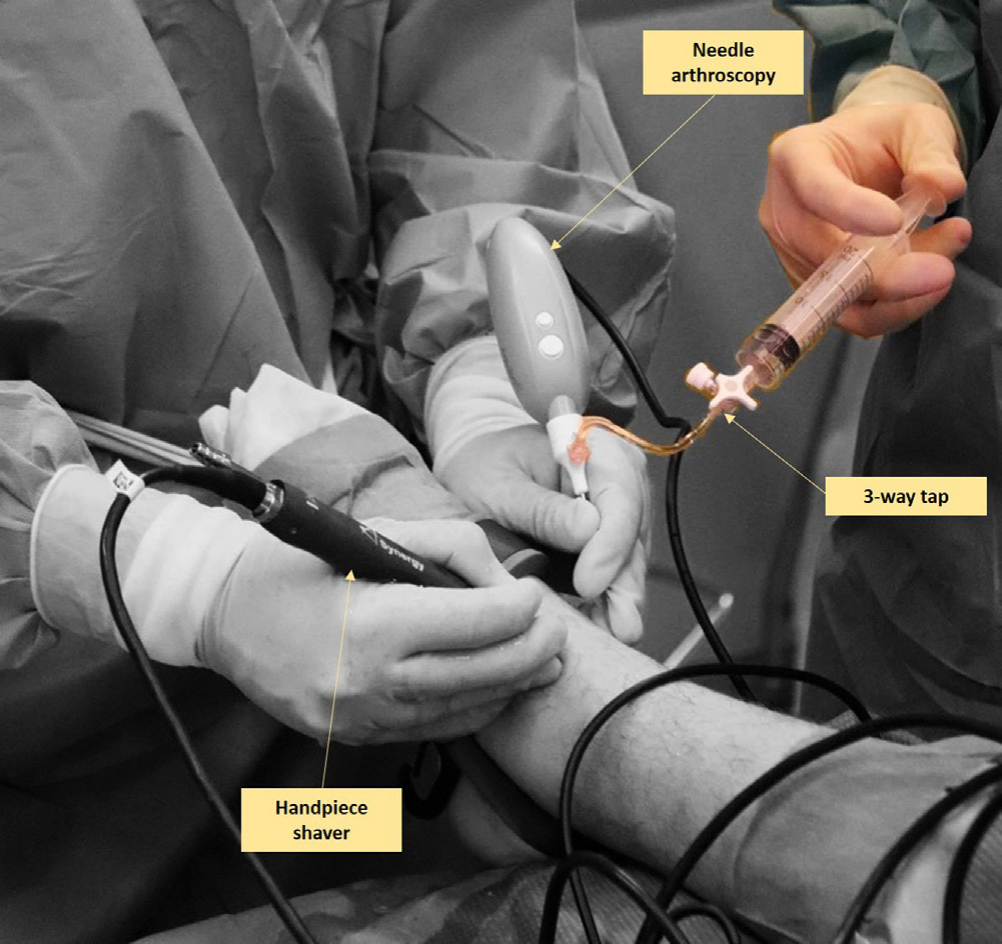
Needle arthroscopic approach to the left ankle via the anterolateral and medial portals. The anteromedial portal is used as primary vision portal, and the anterolateral portal is used as primary intervention portal. A syringe is connected to a 3-way tap—which is connected to the sheath—to distend the joint with sterile saline solution. The saline solution can be injected by an assistant surgeon. In the anterolateral portal a 2 to 3 mm shaver is inserted. This shaver is used to dissect soft-tissue and allow for adequate visualization of the ankle joint.

Portal placement is critical for ensuring the optimal vision of the ankle. Two standard ankle arthroscopic portals, anteromedial and anterolateral, are created.^7^ The anteromedial portal is created first and is used as the primary vision portal. This portal is located on the soft spot just medial to the anterior tibial tendon and the anterior joint line. It is performed with the joint in maximal dorsiflexion to protect the cartilage on introduction. A 2 mm skin incision is created, a sheath with a 2.2 mm diameter is loaded with a blunt obturator, and this sheath is inserted intra-articularly via the skin incision and joint capsule. The obturator is then removed from the sheath and replaced with the needle arthroscope. A syringe is connected to a 3-way tap—which is connected to the sheath—to distend the joint with sterile saline solution (Fig 1).

### Inspection

**Fig 2:**
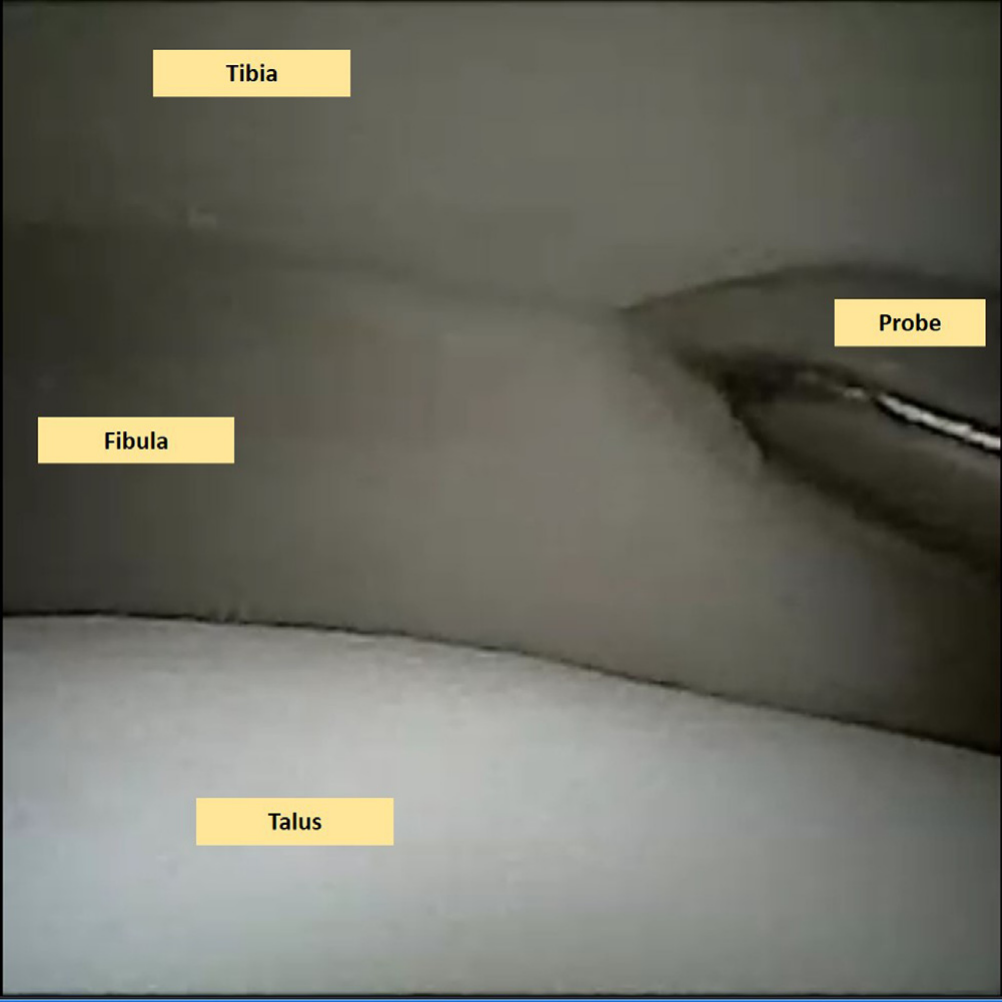
This is a needle arthroscopic view of the left ankle. A thorough examination of the joint takes place including an examination of the instability of the syndesmosis. The fibula is seen on the left side of this image (lateral), the talus on the lower side (distal), and the tibia on the upper (proximal side). The syndesmotic instability is confirmed by applying intra-articular pressure with the probe into the syndesmosis, as well as performing a dynamic extra-articular external rotation stress test.

When the needle arthroscope is inserted into the ankle joint, a step-by-step examination of the joint is performed in each accessible compartment.^3^^,^^8^ The instability of the tibiofibular syndesmosis—which was already diagnosed on a weightbearing computed tomography scan—is examined and confirmed intra-articularly with a probe and through the external rotation test (Fig 2, Video 1). The cartilage layer is assessed in all compartments to determine any kind of lesions and is graded according to the recommendations of the International Cartilage Repair Society on the 9-grid scheme.^9^

### Second Portal and Intervention

**Fig 3:**
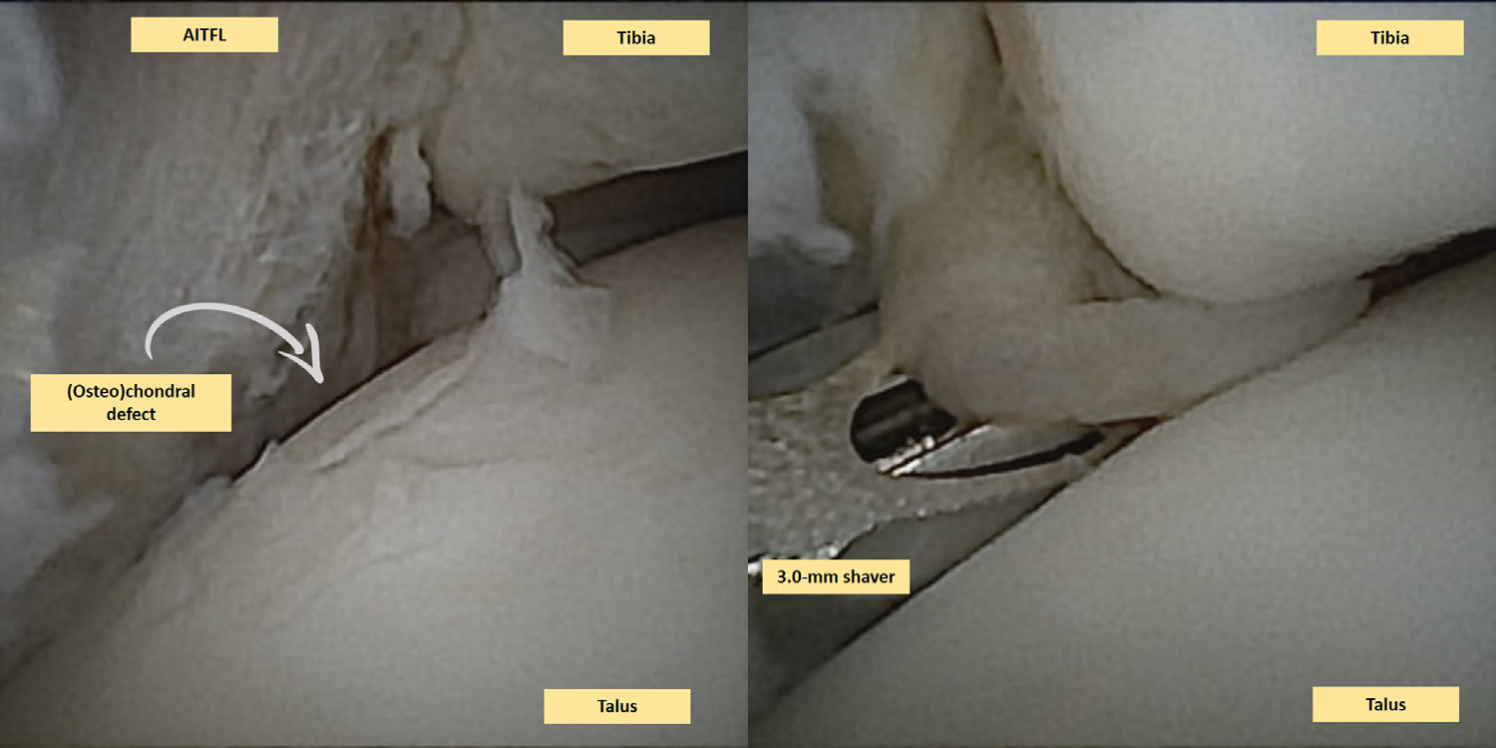
This is a needle arthroscopic view of a right ankle. Two images are included in this figure. The left figure shows a clear (osteo)chondral defect, which is identified and thereafter graded as an International Cartilage Repair Society (ICRS) grade 2. The (osteo)chondral lesion is locate on the lateral talar dome (centroposteriorly). In the right image one can see the hypertrophic anterior inferior talofibular ligament (AITFL) which is also observed on the left image. The hypertrophic ligament causes impingement on the anterolateral ankle joint, and was resected with a 3.0 mm shaver. Moreover, debris and synovial hyperplasia can be resected with the 3.0 mm shaver.

**Fig 4:**
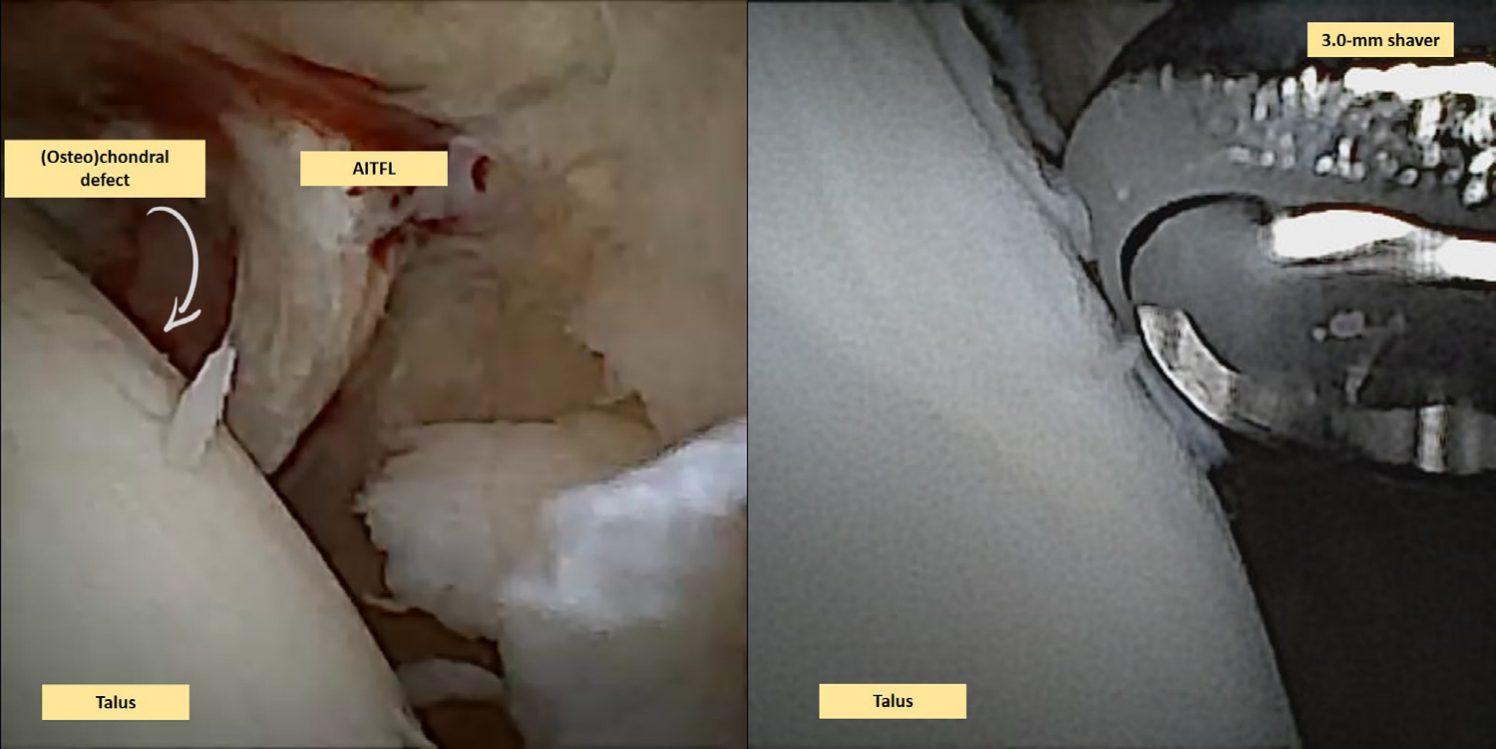
This is a needle arthroscopic view of a left anterior ankle. Because of impact axial trauma, a rupture of the anterior inferior talofibular ligament (AITFL) can be observed in the left image. This is clearly seen because there is a hematoma surrounding the AITFL. Moreover, a clear (osteo)chondral lesion, which is also located on the centroposterior lateral talar dome, can be observed, which was graded as an International Cartilage Repair Society (ICRS) grade 1. On the right image, one can appreciate a 3.0 mm shaver being used to debride the (osteo)chondral lesion and some loose chondral flaps floating intra-articularly.

When intra-articular pathologies (e.g., [O]CLs, loose bodies, or impingement; Figs 3 and 4) are detected, a second—anterolateral—portal is made, using a similar technique (i.e., a skin stab incision and a sheath with a blunt obturator are used to enter the joint capsule). This portal is regarded as the intervention portal for instrument introduction and is positioned just lateral to the peroneus tertius or extensor digitorum longus tendons.^7^^,^^8^ A 2 or 3 mm shaver or burr is introduced into the joint, without the sheath, through the skin stab incision and joint capsule (Figs 3 and 4; Table 1). After the removal of any loose chondral flaps/bodies, the lesion is debrided until the healthy margins of the surrounding cartilage are reached (Fig 4). Soft tissue impingement, hyperplastic synovium, cicatrized joint capsule, and osteophytes may be resected in a concomitant fashion.

### Suture Button, Post-Inspection, Closure, and Postoperative Protocol

**Fig 5:**
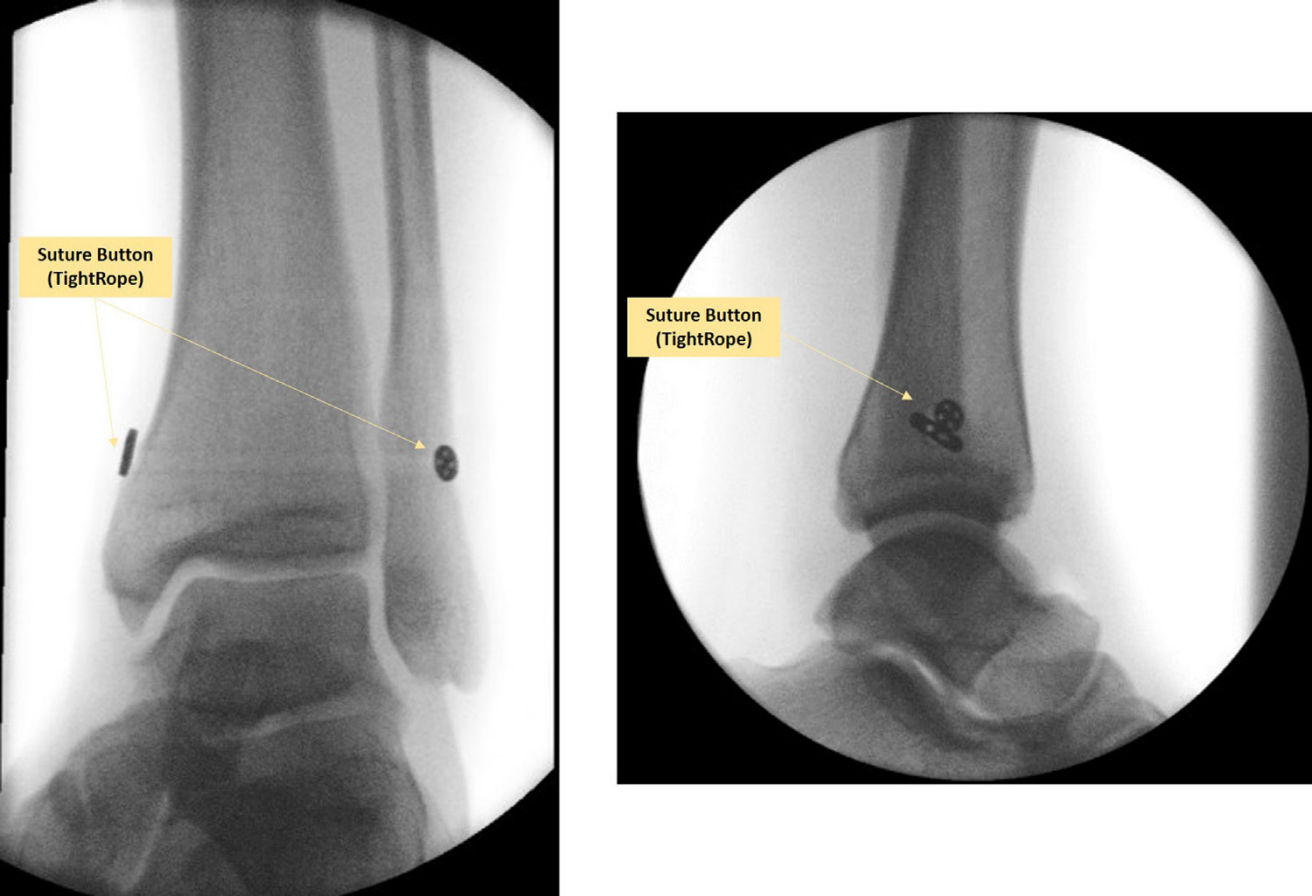
One can observe an anterior-posterior and lateral view of intraoperative fluoroscopy images after the placement of the suture button of a left ankle. The left image shows the anteroposterior view with a clear adequate reduction of the syndesmotic joint, and the right image shows the lateral intraoperative view.

**Fig 6:**
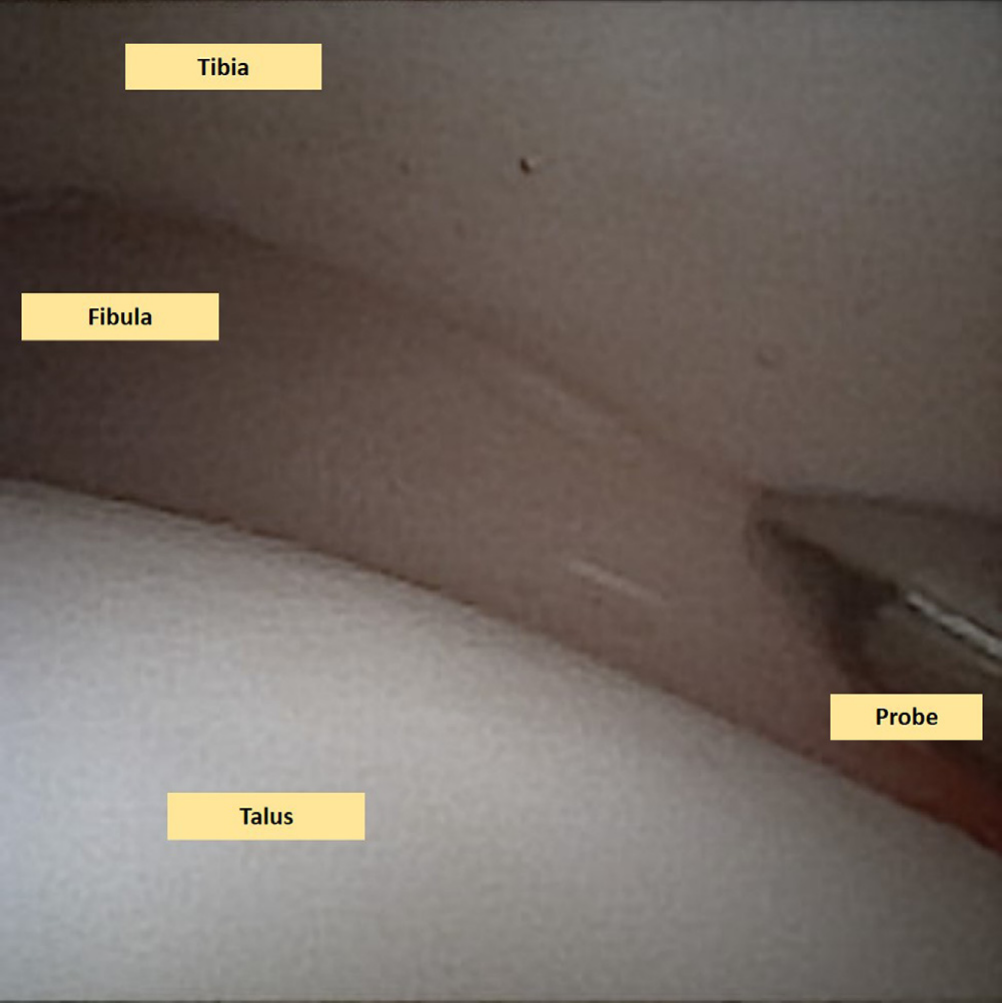
This is an anterior ankle needle arthroscopic view of the left ankle. One can observe that the syndesmotic joint has been stabilized after suture button fixation. The adequateness of the stabilization is confirmed by applying intra-articular pressure with the probe into the syndesmosis; hence, confirming an adequate stabilization. The dynamic extra-articular external rotation stress test can also be repeated to confirm stabilization.

The distal tibiofibular syndesmosis is stabilized by a suture button (Fig 5). An intra-articular post-inspection is performed with needle arthroscopy to confirm a stabilized syndesmosis (Fig 6; Video 1). If needed, a final lavage can be performed to clear any debris. The joint is then aspirated to dryness, and all instruments are removed. Sterile wound closure strips can be used for wound closure of the needle arthroscopic portals.

Closure of the medial and lateral incisions for the placement of the suture button are closed in a layered fashion—subcutaneously with Vicryl 3-0, and the skin itself with Ethilon 3-0. Thereafter, a nonweightbearing posterior ankle splint is applied and replaced by a removable plaster for another week to allow nonweightbearing range of motion exercises. Two weeks after surgery, the removable splint is replaced by a Walker boot; in the Walker boot, the patient is encouraged and advised to progressively initiate weightbearing of the ankle as tolerated.

## Discussion

Both acute and chronic syndesmotic injuries can be associated with intra-articular pathologies. Having a common denominator of an acute traumatic injury being external rotation and forced dorsiflexion, an unstable syndesmotic injury should urge physicians to search for concomitant (osteo)chondral ankle lesions. A recent study indeed showed that (OC)Ls of the ankle are present in at least 20% of these patients.^2^ This number may even be substantially higher in practice, because a lack of standard arthroscopic inspection results in underestimation. Furthermore, early diagnosis may prevent further damage to the joint through lifestyle changes and increase the chances of successful conservative or surgical treatment, potentially reducing the progression toward end-stage osteoarthritis.^10^ In the present Technical Note, we present a needle arthroscopic approach that facilitates an easy and minimally invasive diagnosis and treatment of concomitant intra-articular (OC)Ls before the suture button fixation of the unstable syndesmotic injury.

Using needle arthroscopy in these patients has several advantages. First, the preoperative magnetic resonance imaging scan may not always be able to pick up the presence of small (OC)Ls.^11^ Second, needle arthroscopy allows for a direct and concomitant interventional strategy to damaged cartilage, as well as unstable cartilaginous flaps, or impingement, which can be removed, debrided, and cleaned with the usage of a 2 or 3 mm diameter shaver blade/burr. Third, the diagnosis of an unstable acute or chronic syndesmotic injury can be (dis)confirmed in a dynamic matter as described above.^12^ As a conclusive remark here, one could state that the clinical relevance of adding a needle arthroscopic assessment and concomitant treatment option to a stabilization procedure for an unstable syndesmotic joint is 2-fold. First, it provides an immediate treatment option for concomitantly present cartilage lesions of the ankle. Second, it provides the patient and the treating physician with accurate and increased knowledge of the prognosis of the ankle joint, even in the absence of any cartilage damage.

When considering needle arthroscopy for patients undergoing suture button fixation for unstable syndesmotic injuries, it is important to consider potential pitfalls and disadvantages (Table 2). The 0° inclination—as opposed to the 30° inclination in conventional arthroscopy—may result in a more difficult joint overview of the joint necessitating a learning curve for unfamiliar surgeons. Moreover, it should be stated that the needle arthroscopic procedure may be hampered by a variety of reasons, such as osteoarthritis of the joint, poor vascular status, soft-tissue compromise, and large osteophytes preventing an adequate introduction of the scope.

**Table 2:** Pearls and Pitfalls

Pearls
The needle arthroscopic procedure provides a minimally invasive alternative to conventional arthroscopy for the concomitant diagnostic approach and treatment of (osteo)chondral injuries of the ankle joint associated with syndesmotic injuries warranting a stabilization procedure through suture button fixation, as such enhancing the postoperative rehabilitation and joint stiffness.
The needle arthroscopic inspection and assessment of any intra-articular cartilaginous damage allows for an appropriate localization of the lesion, as well as size determination of the lesion. The presence of the lesion may not always be determined by preoperative magnetic resonance imaging scanning.
Careful drilling and subsequent placement of the suture button system parallel to the ankle joint with the usage of dynamic intraoperative fluoroscopy; the suture button system should be placed from laterally to medially in an anterior fashion (20°-30° anteriorly) with the ankle in dorsiflexion.
Careful placement of the lateral portal under direct arthroscopic visualization.
Decreased postoperative pain levels because of minimal invasiveness of the procedure.
Pitfalls
Incorrect portal placement, causing iatrogenic (neurovascular) damage to the peroneal superficial nerve.
The placement of a suture button system may be associated with postoperative dorsiflexion limitations.
There should be caution not to damage the vena saphena magna when drilling from the fibula toward the medial side of the tibia.
To avoid postoperative stiffness of the ankle joint, prolonged postoperative immobilization should be avoided.
Iatrogenic damage to the articular cartilage of the ankle joint from (incorrect) trocar placement.

In conclusion, we presented a minimally invasive approach to needle arthroscopic diagnosis and treatment of (osteo)chondral injuries of the ankle that may be concomitantly present in patients undergoing suture button fixation for unstable acute or chronic syndesmotic injuries.

## Supplementary Data

ICMJE author disclosure forms

Video 1 The authors describe the technique for needle arthroscopic inspection and treatment of (osteo)chondral lesions of the ankle joint in unstable syndesmotic injuries treated with suture button fixation as a standardized approach. In the video the equipment is noted including sterilization, the set of the needle arthroscopy including a handpiece set, a portable console, 50 mL syringes, and a 3-way tap, as well as sterile sodium chloride 0.9%. Besides the needle arthroscopic set, a needle arthroscopic shaver is used and sterile wound closure strips as well as a compression bandage are used. The video also consist of the set-up; the patient is positioned in a supine manner after which the unstable syndesmotic injury is stabilized with the suture button fixation technique. During the technique, (osteo)chondral lesions are identified and treated concomitantly when necessary.
